# PERSONALITY TRAITS, SCREEN TIME, AND MOBILE PHONE DEPENDENCE ACROSS THE LIFESPAN

**DOI:** 10.1093/geroni/igae098.3686

**Published:** 2024-12-31

**Authors:** Stephanie Washburn, Hailey Andrews, Nelson Roque

**Affiliations:** University of Florida, Gainesville, Florida, United States; Pennsylvania State University, University Park, Pennsylvania, United States; Pennsylvania State University, University Park, Pennsylvania, United States

## Abstract

Digital technologies have become integral to daily life worldwide, with many individuals increasingly dependent on mobile devices like smartphones and tablets. The widespread growth of technology use worldwide highlights the need to understand how individual traits contribute to mobile device dependence and its potential impacts on mental health, paving the way for interventions to mitigate these effects. This study examined the association between self-reported screen time, mobile phone dependence (Nomophobia Questionnaire), and personality traits (Ten Item Personality Inventory), across the lifespan. Data were collected from 550 adults (Mage=39yrs, Rangeage=19-77; 54% female; 59% non-Hispanic White) through an online survey, delivered on Prolific. Linear regression models, adjusted for age and gender, assessed the relationship between personality traits and two outcomes: self-reported screen time and mobile phone dependence. For screen time (N = 299), significant predictors were age (β = -0.06, p =.001), conscientiousness (β = -0.44, p =.013), and agreeableness (β = -0.47, p =.014). For mobile phone dependence (N = 550), significant predictors were age (β = -0.33, p =.001), emotional stability (β = -5.07, p <.001), and agreeableness (β = 2.59, p =.021). These results suggest that three of the Big Five personality traits influence screen time and mobile phone dependence. Understanding these factors may guide interventions to reduce dependency and its mental health effects. Future longitudinal studies are needed to explore these relationships over time and establish causality.

